# Host genotype and environment affect the trade-off between horizontal and vertical transmission of the parasite *Edhazardia aedis*

**DOI:** 10.1186/s12862-018-1184-3

**Published:** 2018-04-24

**Authors:** Giacomo Zilio, Kevin Thiévent, Jacob C. Koella

**Affiliations:** Institute of Biology, University of Neuchâtel, Rue Emile-Argand 11, 2000 Neuchâtel, Switzerland

**Keywords:** Mixed-mode transmission, Trade-off, Genetic correlation, Parasite, Virulence

## Abstract

**Background:**

If a parasite is able to transmit horizontally or vertically, which transmission mode will it choose? We investigated how the growth conditions and the genotype of the mosquito *Aedes aegypti* affect the transmission mode of the parasite Edhazardia aedis.

**Results:**

In poor conditions the parasites were more likely to be transmitted horizontally, whereas in favourable conditions they were more likely to be transmitted vertically. Unfavourable conditions delayed emergence, giving the parasite more time to produce its horizontally transmitted stage; in more favourable conditions mosquitoes have greater reproductive success, increasing the effectiveness of vertical transmission. In addition, the parasite’s ability to transmit vertically was influenced by the genetic background of the host (i.e., its full-sib family), giving a genetic correlation between the host’s life-history and which of the parasite’s transmission mode it enables. In particular, genotypes with large bodies (and therefore high fecundity) were more likely to enable vertical transmission than genotypes with small bodies. This led to a trade-off among the host’s families (which can be interpreted as a genetic correlation) for the parasite’s transmission mode.

**Conclusions:**

Since horizontal transmission is linked to higher virulence than vertical transmission, the host’s contribution to transmission mode has important consequences for the evolution of parasites with mixed-mode transmission.

## Background

The rate of transmission and the severity of symptoms (i.e. virulence) determine a parasite’s damage to a population of hosts, and they are the center of most modern ideas about the evolution of parasites [1, 2]. Ideas about virulence, for example, are often based on a link between virulence and rate of transmission, and explain virulence as an unavoidable side-product of selection for more transmission [3]. Virulence is also predicted to change according to the mode of transmission. Horizontal transmission can be associated with high virulence, if the strong exploitation of the host increases the rate of transmission. In contrast vertical transmission is expected to be associated with low virulence, for the parasite and the host share to a large degree their evolutionary interests [4].

While the role of transmission mode on the evolution of virulence is often studied, relatively little attention has been given to how transmission mode evolves [5, 6]. Theoretical exceptions predict the conditions that enable vertical and horizontal transmission to coexist and their resulting levels of virulence [7, 8]. These models can be applied to many parasites with mixed modes of transmission such as the medically important viruses HIV, human papilloma virus, and hepatitis B and C viruses.

In many parasites with such mixed-mode transmission, the two transmission modes are physiologically, developmentally or evolutionarily linked [9–12]. In many cases, this link means that transmitting with one mode, either vertically or horizontally, reduces transmission with the other [6], but vertical and horizontal transmission can also be positively correlated [13–15]. When a trade-off between the two transmission modes is present, it strongly influences the parasite’s evolution and dynamics [7, 16]. For example, the nature of virulence of the horizontal component (additional mortality or reduced fecundity in the host) may impose constraints on the shape of the trade-off. Only a convex shape of the trade-off will allow the coexistence of the two transmission modes, while a concave one will lead to the fixation of either one or the other [8]. This implies that the trade-off between the two transmission modes is an important part of a parasite’s epidemiology [17].

In most cases we know little about what controls this trade-off, and in particular about the genetic basis of the trade-off. Although several studies have measured the trade-off between horizontal and vertical transmission, almost all of them have focused on the variation due to the environment rather than to the host’s or parasite’s genotypes. One example is the bacterium *Holospora undulata* that infects the ciliate *Paramecium caudatum*. The bacterium has a reproductive stage that is used to infect the daughter nuclei of infected cells, and it has an infectious stage that can be transmitted horizontally. If the system is maintained in a situation enabling high replication rates of the host, the parasite remains mainly in the reproductive stage and is passed on vertically to the daughter cells of mitotically dividing paramecia; in contrast, at low population growth rate, the parasite differentiates into infectious forms and is transmitted horizontally [18]. A second example is the microsporidian *Edhazardia aedis* that infects the mosquito *Aedes aegypti*. The parasite has two forms of spores: binucleate spores that infect the eggs of infected females and are transmitted vertically, and uninucleate spores that can kill infected larvae and are transmitted horizontally among larvae. There is an obvious trade-off in this system: horizontal transmission requires the death of larvae, so that it precludes vertical transmission. The investment in the two spore types is regulated in a way that horizontal transmission becomes more likely as food conditions deteriorate and therefore larvae grow more slowly [19, 20]. An adaptive explanation is that only good food conditions enable the host to lay many eggs and therefore the parasite to have efficient vertical transmission; the potential for vertical transmission decreases as larval growth slows, making the mosquitoes more attractive to exploitation for horizontal transmission [19, 20].

Such an adaptive explanation requires that the transmission mode is ultimately controlled by the parasite’s genes. Parasitic control has indeed been shown in a number of cases. Thus, differences among strains of the fungus *Atkinsonella hypoxylon* differ in the production of the fruiting bodies responsible for horizontal transmission [14]. Moreover, manipulating levels of horizontal and vertical transmission have led to evolutionary changes of the pathogen’s transmission mode in several studies, for example bacteriophages [21], barley stripe virus infecting *Hordeum vulgare* [22], and the bacterium *Holospora undulata* infecting *Paramecium caudatum* [11]. While there are thus a few suggestions of the parasite’s genes control of its transmission mode, we may expect that the transmission mode and the trade-off between horizontal and vertical transmission are also influenced by the host’s genes. Thus, selection for delayed pupation of the mosquito brings with it more vertical and less horizontal transmission of *E. aedis*. [20]. Furthermore, several traits of host-parasite interactions are governed by a combination of host and parasite genotypes [23–26]. The interaction between host and parasite genotypes govern, for example, the variability of the virulence and transmission of *Holospora undulata* [26], and the production of the fruiting bodies of the fungus *Epichloë elymi* depends on the genotype of the grass *Elymus hystrix* on which the fungus is growing [27]. A different genetic background of a parasite’s host may therefore promote divergent evolutionary trajectories for the parasite, for each host will cause a particular selection pressure on the parasite. Parasites infecting populations of hosts with different genetic background may therefore experience different selection pressures. Such a host-parasite interaction may consequently shape spatial gradients of local adaptation in accordance with the idea of the geographic mosaic of co-evolution [28, 29].

In this study, we investigated how the host’s genotype and food environment interact to influence the horizontal and vertical transmission of *E. aedis.* We expected that lower food availability would favour horizontal over vertical transmission. We further expected that the mosquito’s genotypes enabling large size and long life would favour vertical transmission, while genotypes with a long development would favour horizontal transmission, and that these associations would be more apparent when food is scarce. Finally, we expected to find a trade-off in the host’s potential to transmit the parasite horizontally or vertically.

## Methods

### Mosquitoes

*Aedes aegypti*, a mosquito that occurs throughout the tropics and subtropics, is the main vector of yellow, dengue and chikungunya fevers. Its physiology, genetics, and ecology have been intensively studied [30, 31]. The larvae of *Ae. aegypti* grow in natural or artificial containers and their development depends on many environmental factors [32]. Two aspects that make it suitable for lab work is that its eggs can be stored for a long period and that a single mating lets the female lay eggs throughout her life [33, 34]. We used the UGAL strain of the mosquito *Ae. aegypti* (obtained from P. Guérin, University of Neuchâtel), an undocumented strain that was established in the 1970s [35]. The mosquitoes were maintained at 26 °C, 70% humidity, 12 h:12 h light:dark photoperiod.

### Microsporidia

**Fig. 1:**
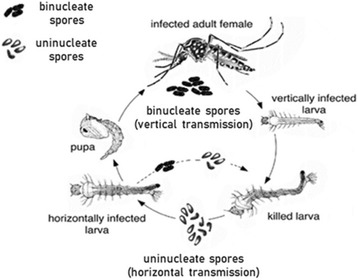
Life cycle of the microsporidia *Edhazardia aedis* infecting the mosquito *Aedes aegypti*. Usually (solid line), the parasite alternate vertical and horizontal transmission using two types of spores. Repeated horizontal transmission is possible (dashed line). Since the parasite’s life-cycle involves a strict alternation of binucleate and uninucleate spores, repeated horizontal transmission implies that the parasite goes through its complete developmental sequence – producing first binucleate and the uninucleate spores – within a juvenile mosquito. (Modified from [42])

*Edhazardia aedis* was provided by J. J. Becnel at the United States Department of Agriculture (Gainesville, Florida). This microsporidium, a specific parasite of *Ae. aegypti*, has a complex life cycle (Fig. 1) involving horizontal and vertical transmission [36–40] and using two types of morphologically distinguishable spores: uninucleate spores for horizontal transmission and binucleate spores for vertical transmission. Mosquito larvae are infected when they ingest uninucleate spores suspended in water. After a period of development the parasite produces binucleate spores in adult females; these are responsible for vertical (transovarial) transmission. Males provide no opportunity for vertical transmission, so adult males are a dead-end for the parasite. Once the infected eggs hatch the parasite develops into uninucleate spores. These vertically infected mosquitoes die as juveniles and release the uninucleate spores into the water. These uninucleate spores are eaten by larvae to complete the life-cycle. Thus, larvae that acquire the spores horizontally usually go on to transmit vertically, while larvae that are infected vertically die as larvae. Occasionally, as illustrated in Fig. 1, horizontally infected larvae die before emergence, which can result in a second round of horizontal transmission without the requirement of vertical transmission [36]. Note that underlying this sequence of transmission is a fixed developmental sequence (though not necessarily fixed timing) of the parasite that alternates the production of binucleate and uninucleate spores [36, 38]. Thus, if the horizontally infected individuals die for a second round of horizontal transmission, the parasite has switched from uninucleate to binucleate and then back to uninucleate spores.

### Experimental design

Our main goal was to see how the host’s genotype and environment affect the developmental trade-off between horizontal and vertical transmission. We approximated genetic variation with the variation among full-sib families, where we minimized the maternal effects by rearing the mosquitoes of the parental generation individually in identical environments. We chose as environmental factor the amount of food given to the larvae. We exposed the larvae to a standard concentration of uninucleate spores, and then followed the mosquito and parasite’s development to evaluate the potential for horizontal and vertical transmission.

#### Full-sib families

In order to synchronize the hatching and thus obtain larvae of the same age, we hatched uninfected eggs from the colony at low air pressure. The larvae were moved to and individually reared in 12-well tissue-culture plates filled with 3 mL of deionized water. They were fed daily with TetraMin™ fish food (age 0 (day of hatching, day 0): 0.06 mg/larva, age 1: 0.08 mg, age 2: 0.16 mg, age 3: 0.32 mg, age 4: 0.64 mg, from age 5 onwards: 0.32 mg). Each pupa was placed into a 180-mL plastic cup covered with bed-netting. Three days after emergence, two males were transferred into each of 100 cups all containing one female. Two days later, the males were removed, the females were allowed to blood feed on GZ’s arms for 10 min, and then given the opportunity to lay eggs on a filter paper for 5 days. The filter paper with the eggs was then stored in a petri dish at the same laboratory conditions as the colony. The cycle of blood-feeding and egg-collecting was repeated for 10 weeks. During this period, adults were also provided with a cotton ball soaked with 10% sugar solution, which was changed every 2 days. All the eggs collected from one female during her lifespan represented a full-sib family (since females only mate with one male), for a total of 100 full-sib families.

#### Main experiment

The eggs were gently brushed into petri dishes and rehydrated in deionised water. Thirty of hundred families were synchronously hatched under partial vacuum, twelve of the families with enough larvae were haphazardly chosen for the experiment. The larvae were reared individually in 12-well tissue-culture plates filled with 3 mL of deionized. Each plate contained an individual from each family.

Each family experienced two larval food treatments (50% or 100% of our standardized ration of TetraMin Baby™ fish food (see above)).

Seventy-two hours after hatching, the larvae were exposed in their wells to 500 uninucleate spores. The spores had been harvested from vertically infected larvae hatched 7 days earlier than the day of infection. Twenty of these vertically infected larvae were crushed and homogenized in an Eppendorf tube adding 1 mL of deionized water, and the concentration of the uninucleate spores was determined with a hemocytometer and a phase-contrast microscope (Zeiss Axio Lab.A1).

Pupae were individually transferred to Falcon tubes, and the emerging adults were provided with a cotton ball soaked with 10% sugar solution that was changed every 6 days. The survival was checked daily. The dead larvae, pupae and adults were stored in 2 mL plastic tubes at − 20 °C until further investigation.

We measured the spore load for each of the collected individuals after adding 0.1 mL of deionized water and homogenizing the samples with a TissueLyser LT - QIAGEN. The numbers of uninucleate and binucleate spores in the obtained solution were counted with a hemocytometer placed under a phase contrast microscope (Zeiss Axio Lab.A1).

The treatment conditions and the family origin of the samples were unknown during the counting of the spores, a total amount of 1904 individuals were used for the analysis [41] with a median of 155 individuals per family.

### Statistical analysis

We considered that mosquitoes enabled horizontal transmission, if they died before emerging and harbored uninucleate spores, and that they did not enable horizontal transmission either if they carried no uninucleate spores or did not die as juveniles. We considered that mosquitoes enabled vertical transmission, if adult females carried binucleate spores, and that they did not enable vertical transmission otherwise. Note that we used these qualitative measures of transmission rather than quantitative measures involving the number of spores for two reasons. First it is not known how the number of spores relates to transmission. Second, most of the mosquitoes do not carry any spores (in particular uninucleate spores), so that any quantitative measure would strongly resemble the qualitative one.

We first evaluated the effects of food, full-sib family and their interaction on each transmission mode with a generalized linear mixed effect model with binomial distribution and on age at pupation, wing length and longevity with an ANOVA. For each trait we considered food treatment and family as fixed factors. If the interaction was not significant, we show the results of the analysis that includes only the main effects. Note that we considered family as fixed rather than random, because we were not interested in the variation among families within our colony. Rather, we wanted to check for differences among the families of our study that we could then analyze in the next step of the analysis.

#### Genetic correlation and trade-off

The potential genetic correlation between transmission success (proportion of individuals enabling horizontal or vertical transmission) and life-history traits was analyzed with regressions of the family means. We looked at the correlation with juvenile developmental period (days spent in the aquatic environment before emergence as an adult), adult size (wing length), and longevity after emergence in the different food treatments.

We investigated a possible trade-off between vertical and horizontal transmission with a linear regression with the family-means of the horizontal and vertical transmission at each food condition.

Finally, we evaluated the correlation of vertical transmission of the families between the two food regimes with a linear model. (The analogous analysis for horizontal transmission would be meaningless, for almost all families allowed no horizontal transmission in the good-food environment.)

All the statistical analyses were performed with R version 3.4.0.

## Results

### Effects of food and family on transmission

Families ranged from 11% survival up to emergence (at low food) to 97% (at high food).

**Fig. 2:**
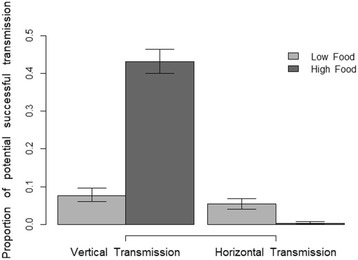
Proportion of potential for vertical and horizontal transmission for low and high food conditions. The bars representthe proportion (± 95% confidence intervals) of the individuals enabling horizontal or vertical transmission according to their developmental stage (adults harbouring binucleates spores for vertical and juvenile harbouring uninucleate spores for horizontal transmission)

The effect of food was highly significant for the potential horizontal and vertical transmission. Although 9% of the mosquitoes that fed the high-food diet and 76% of the low-food mosquitoes died before emerging, only 2% and 6% of these, respectively, harbored uninucleate spores. The potential for horizontal transmission (the proportion of mosquitoes that died before emergence and harboured uninucleate spores) at high and low food was thus 0.2% and 5% of the exposed individuals (χ^2^ = 54.1, df = 1, *p* < 0.001) (Fig. 2). Among the mosquitoes that emerged as adult females, 91% and 95% harboured binucleate spores at high and low food, and respectively 42% and 7% of the these females enabled vertical transmission (χ^2^ = 350, df = 1, *p* < 0.001) (Fig. 2).

Families ranged from 0% (at high food) to 15% (at low food) horizontal transmission and from 1% (at low food) to 54% (at high food) vertical transmission. The effect of the full-sib families was significant for both the vertical (χ^2^ = 29.4, df = 11, *p* = 0.002) and the horizontal transmission mode (χ^2^ = 33.5, df = 11, *p* < 0.001). The interactions between food and family (so, whether the effect of food on the success of the two transmission modes depended on the full-sib families) was significant for vertical transmission (χ^2^ = 25.7, df = 11, *p* = 0.007) but not for horizontal transmission (χ^2^ = 5.6, df = 11, *p* = 0.90).

### Effects of food and family on life history traits

Average age at emergence ranged from 6.2 for one of the families reared with high food to 9.7 days, with highly significant effects of family (F_11,990_ = 28.3, *p* < 0.001) and food level (F_1,990_ = 222, *p* < 0.001). Average wing length ranged from 2.82 mm at high food to 2.32 mm at low food (family: F_11,990_ = 3.34, p < 0.001; food: F_1,990_ = 432, *p* < 0.001). Average longevity after emergence ranged from 12 days at high food to 0.6 days at low food (family: F_11,990_ = 2.08, *p* = 0.019; food: F_1,990_ = 160, *p* < 0.001).

### Genetic correlations of transmission and life-history

**Fig. 3:**
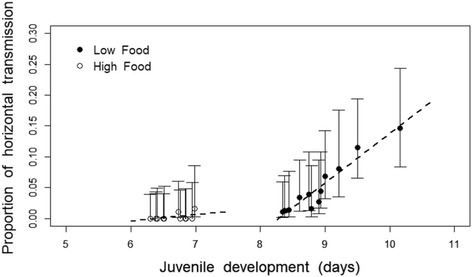
Relationship between horizontal transmission and juvenile development. Dots represent the proportion of horizontal transmission per family (proportion of individuals whithin the family enabling horizontal transmission) as a function of the time spent as juvenile at low (closed symbols) and high food (open symbols). The lines represent the regression of the linear model for each food treatment and the bars the±95% confidence intervals

The proportion of the family that enabled horizontal transmission (proportion of individuals within the family enabling horizontal transmission) was higher in low food than in high food conditions (F = 35.5, *p* < 0.001) and increased with the average juvenile developmental period of the family (F = 36.5, *p* < 0.001), as shown in Fig. 3. There was a tendency for the relationship between the family’s potential for horizontal transmission and juvenile development to be affected by food (food* juvenile development: F = 3.51, *p* = 0.098). Neither body size (F = 1.4, *p* = 0.27), longevity (F = 0.4, *p* = 0.556) or other interactions (all *p*-values > 0.13) affected the family’s potential for horizontal transmission.

**Fig. 4:**
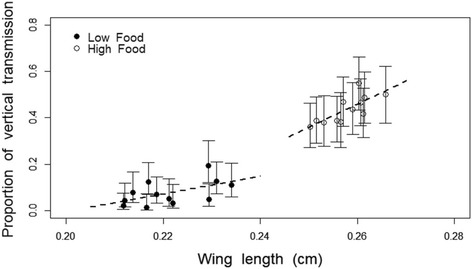
Relationship between vertical transmission and wing length (proxy for adult size). Dots represent the proportion of vertical transmission (proportion of individuals whithin the family enabling vertical transmission) as a function of the size of the wings at low (closed symbols) and high food (open symbols). The lines represent the regression of the linear model for the two food treatments and the bars the±95% confidence intervals

The proportion of the family that enables vertical transmission (proportion of individuals within the family enabling vertical transmission) was higher in high food than in low food conditions (F = 96.8, *p* < 0.001) (Fig. 4). It increased with mean body size (F = 29.8, *p* < 0.001) and longevity (F = 5.3, *p* = 0.049) and it decreased with juvenile developmental period (F = 8.1, *p* = 0.022). Food modified the effect of body size (food*body size: F = 13.5, *p* = 0.006) and the interaction between mean juvenile development and longevity (juvenile developmental period*longevity: F = 5.8, *p* = 0.043; food*juvenile developmental period*longevity: F = 6.5, *p* = 0.033). In addition, there was a tendency for an interaction between mean body size and mean juvenile development to affect a family’s potential for vertical transmission (F = 4.3, *p* = 0.071), but no other interactions were significant (all *p*-values > 0.28).

### Trade-off between vertical and horizontal transmission

**Fig. 5:**
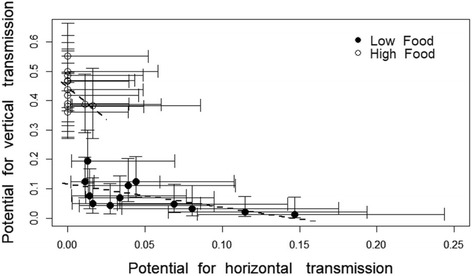
Potential for vertical transmission as a function of the potential for horizontal transmission in *Edhazardia aedis* at low food (closed symbols) and high food (open symbols). Each symbol represents the proportion of individuals within the family enabling vertical and horizontal transmission. The lines show the regression of the means, the bars represent the±95% confidence intervals

The mean potential within families for the parasite to be transmitted vertically was negatively correlated with the average potential of horizontal transmission (F = 23.1, df = 1, r^2^ = 0.49, *p* < 0.001) (Fig. 5). Although food treatment had no significant effect on the trade-off between vertical and horizontal transmission (F = 1.19, *p* = 0.285), this result is difficult to interpret because of the limited horizontal transmission at the high food level.

### Correlation between environments

**Fig. 6:**
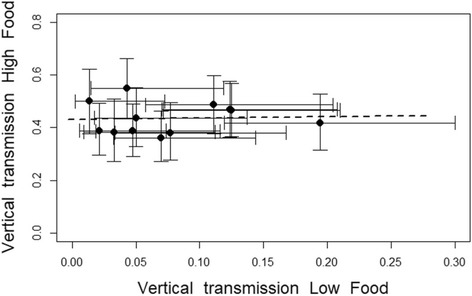
Correlation between the potential for vertical transmission (proportion of individuals whithin the family enabling vertical transmission) in high and low food conditions. Each dot represents the mean per family, the dashed line the regression line of the model and the barsthe ±95% confidence intervals

The potential of a family for vertical transmission was uncorrelated between the two food treatments (F_1,10_ = 0.0291, *p* = 0.868) (Fig. 6). The analogous analysis for horizontal transmission would be meaningless, for almost all families allowed no horizontal transmission in the good-food environment.

## Discussion

Our results confirm that the transmission mode of *E. aedis* is affected by the growth conditions of the host. They also highlight the role of the host’s genotype in linking its life-history to the parasite’s transmission and in influencing the trade-offs underlying transmission mode.

Corroborating earlier studies [19, 20], the parasite shows an adaptive response to changes of the host’s development. Food conditions that slow growth increased the potential for horizontal transmission whereas favorable conditions (high food) increased vertical transmission. If growth is slow, adults are small and have low fecundity. Vertical transmission is therefore inefficient. However, poor food conditions also delay pupation, giving the parasite more time to complete its development from binucleate to uninucleate spores within a single larva. This enables the parasite to achieve a high load of horizontally transmitted spores, and thus to kill its host [20] and being transmitted efficiently. If growth is fast, in contrast, the parasite can expect that its host will lay many eggs; it therefore puts more emphasis on vertical transmission. Even if the rapid development does not allow the parasite to develop many spores, vertical transmission can be efficient, for only a few spores are necessary to infect all of the mosquito’s eggs [37]. Transmission mode thus appears to be controlled by the production of uninucleate spores and the associated risk of larval mortality rather than by different allocation to the production of binucleate spores.

Note that our use of the phrase ‘adaptive response’ refers to (non-genetic) changes of the parasite’s transmission mode. This does not imply that the parasite alters its development. Indeed, at least part of the adaptive response is simply a consequence of the timing of the parasite’s fixed developmental pattern in the host’s development, The parasite cannot produce a new generation of uninucleate spores without first going through a generation of binucleate spores. Uninucleate spores are therefore more likely to be found in more slowly developing larvae. An interesting implication of this idea is that the parasite has evolved its fixed developmental pattern in a way that optimises its transmission in its very variable environment (that is, host).

In addition to the response of the parasite to the host’s environment, we found that the parasite changed its response according to the host’s genetic background. The mosquito families differed with regard to the potential for transmission mode thus showing the presence of a genetic variation in the host for the transmission mode of the parasite. Genetic variation of a host for the transmission mode of a parasite has also been detected in the grass *Elymus hystrix* infected by the endophyte *Epichloë elymi* [27]. In our case, the genetic basis for vertical transmission corroborates an experiment where artificial selection of the mosquito for more rapid or slower development affected the parasite’s transmission mode [42].

Our data go further by suggesting that the parasite’s transmission mode changes according to the genetic basis of several life-history traits such as larval survival, adult size and longevity. These genetic differences in life-history traits represent in themselves a first defence of the host against parasite threats, influencing the transmission mode which determines how parasites infect and exploit the hosts. The variation in the life-history traits of the host therefore has important consequences not only for the parasite but also for host’s fitness. In particular, the families with the largest and longest-living individuals were most likely to enable vertical transmission (low virulence and higher fitness for the host), while the families with the slowest development were most likely to enable horizontal transmission (high virulence and lower fitness for the host). Although molecular and physiological effects were not measured, our results therefore suggest that several life-history traits are genetically linked to the complex infection-related and immunity genes. Such a trade-off between life-history traits and immune response is often assumed [43] and supported by empirical studies [44, 45], including in our host-parasite system [39]. But the results also support the idea that the parasites switch in an adaptive way their transmission mode according not only to the environment, but also to the genotype of the host they are infecting. In families whose genetic background suggests high expected reproductive success, the parasite transmits vertically; in families with low reproductive success and long development the parasite switches to horizontal transmission. This switch according to genetic background is necessary to let the parasite to achieve high success, for the families that permit more vertical transmission permit less horizontal transmission (at least in the low-food conditions, where it was possible to investigate this trade-off).

The trade-off between vertical and horizontal transmission has important consequences for the evolution of, both, the parasite and the host, in particular because horizontal transmission is associated with higher virulence than vertical transmission [46]. For *E. aedis*, this association is necessary because of the parasite’s life-cycle, and more generally this trade-off is predicted by evolutionary theory [7, 8, 16, 47]. First, there should be selection for hosts that enable more vertical and less horizontal transmission. This could lead in the long-term to the loss of horizontal transmission and therefore more effective vertical transmission. Indeed, this has been observed in an evolutionary experiment involving *P. caudatum* and *H. undulata* [12]. Second, since the host’s genetic structure affects the ability of the parasite to transmit horizontally, it also affects the evolution of the parasite’s virulence. In particular, with the expected selection of the host towards less damaging vertical transmission, we would expect a correlated response of the parasite to lower virulence thus reinforcing the evolution to vertical transmission. Third, while at high food hosts develop rapidly and almost always enable vertical transmission, we expect at low food and in the absence of parasite pressure that the host’s life-history evolves to delayed age at maturity [48]. Introducing parasites pressure impedes this evolution towards an optimal strategy by forcing the host towards vertical transmission with early maturity, although the associated low fecundity is not beneficial for either the host or the parasite. Under parasite pressure indeed, slow development hosts are more likely to die, whereas fast development host are favored but with a smaller size than the optimum and consequently with a cost in fecundity.

## Conclusions

We confirmed a response of *E. aedis* in its transmission strategy according to environmental conditions and the host’s genotype. This response may be considered to be adaptive, for it leads to effective vertical transmission when the host is expected to achieve high fecundity and to more horizontal transmission when the host has a slow rate of development. Since mode of transmission is linked to the evolution of the parasite’s virulence, the host’s contribution to the trade-offs underlying transmission can influence considerably the epidemiology and evolution of parasites with mixed-mode transmission.
